# A Gastrointestinal Electrical Stimulation System Based on Transcutaneous Power Transmission Technology

**DOI:** 10.1155/2014/728572

**Published:** 2014-06-25

**Authors:** Bingquan Zhu, Yongbing Wang, Guozheng Yan, Pingping Jiang, Zhiqiang Liu

**Affiliations:** ^1^Institute of Precise Engineering and Intelligent Microsystems, Shanghai Jiaotong University, Shanghai 200240, China; ^2^Department of General Surgery, Shanghai Pudong New Area People's Hospital, Shanghai 201299, China

## Abstract

Electrical stimulation has been suggested as a possible treatment for various functional gastrointestinal disorders (FGID). This paper presents a transcutaneous power supplied implantable electrical stimulation system. This technology solves the problem of supplying extended power to an implanted electrical stimulator. After implantation, the stimulation parameters can be reprogrammed by the external controller and then transmitted to the implanted stimulator. This would enable parametric studies to investigate the efficacy of various stimulation parameters in promoting gastrointestinal contractions. A pressure detector in the internal stimulator can provide real-time feedback about variations in the gastrointestinal tract. An optimal stimulation protocol leading to cecal contractions has been proposed: stimulation bursts of 3 ms pulse width, 10 V amplitude, 40 Hz frequency, and 20 s duration. The animal experiment demonstrated the functionality of the system and validated the effects of different stimulation parameters on cecal contractions.

## 1. Introduction

FGID is a common disease that disrupts patients' daily lives; symptoms include abdominal pain, abdominal distention, diarrhea, nausea, and constipation. The prevalence of FGID has been estimated to range from 23.5% to 74%. Because the etiology of and mechanisms behind the disease are not well understood, an effective treatment has yet to be developed.

Research has found that FGID is always accompanied by gastrointestinal motility disorder (GIMD), so improving gastrointestinal motility has been proposed as a possible treatment. After cisapride, tegaserod, and alosetron were withdrawn from the market, very few prokinetic drugs remained available, and their efficacy in treating severe motility disorders was modest at best. Surgery rarely provides significant symptom relief in patients with generalized gut dysmotility. Thus, alternative interventions have been actively explored. Gastroparesis and severe dyspepsia represent two conditions that affect quality of life, are associated with high medical costs [1, 2], and are often refractory to dietary and pharmacological interventions.

In addition to the application of gastrointestinal kinetic agents, gastrointestinal electrical stimulation (GES) has attracted worldwide attention and has been extensively studied as a potential treatment. In 1963, Bilgutay et al. proposed GES as a treatment for GIMD [3]. In the past two decades, GES research has led to new directions, new fields, and major breakthroughs. The therapeutic effect of GES has been verified in gastroparalysis, constipation, obesity, and others [4–7].

Although extensive research has proved that GES can improve gastrointestinal motility and alleviate the symptoms of FGID, the clinical application of GES is still restricted by three major problems related to existing electrical stimulation devices. One is the limited range of stimulation parameters caused by the finite life span of the power supply [8]. In former studies, a stimulation pulse with an ms-level width is considered to be the most effective in promoting gastrointestinal contractions [9], but no existing implantable stimulation devices can generate electrical pulses with widths above 2 ms [10, 11]. Second, the devices are limited by ineffective methods of monitoring gastrointestinal contractions. Implantable stimulation devices that can record gastrointestinal contractions have not been developed. Finally, limited battery power causes an obvious power-supply problem when the electrical stimulator needs to be permanently implanted. In the light of research on magnetic coupling in recent years [12–14], transcutaneous power transmission has become a feasible solution for this power-supply issue.

To further refine the treatment technology for FGID and advance the clinical application of GES, this paper aimed to develop a complete implantable electrical stimulation system. The specific goals of the present work were (1) to solve the problem of long-term power supply for an implantable electrical stimulator to extend the range of stimulation parameters and (2) to add a pressure-detection function to enable real-time tracking of the effects of GES. Based on the resultant implantable electrical stimulation system, this paper also studied cecum electrical stimulation in animal experiments to investigate the effect of GES on gastrointestinal contractions, in order to lay the groundwork for selection and verification of stimulation patterns for the treatment of FGID.

## 2. Methods and Materials

**Figure 1:**
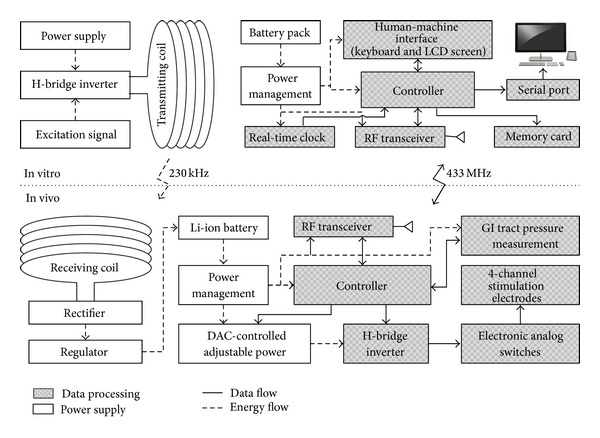
System principle.

The system consisted of two subsystems: the electrical stimulation system and the wireless transcutaneous power supply system. The electrical stimulation system generated various stimulation pulses based on programmed parameters; it then sent the detected pressure to the external controller to compare the measures before and after stimulation. The transcutaneous power supply system charged the implanted electrical stimulator when its battery capacity was low and simultaneously powered the device. The principles behind the system are illustrated in Figure 1.

### 2.1. Electrical Stimulator

**Figure 2:**
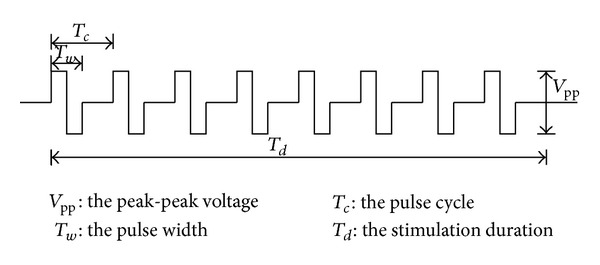
Stimulation pulses.

The electrical stimulator can generate 4-channel adjustable stimulation pulses to act on the GI tract, detect pressure variation, and provide feedback on the effects of the stimulation. The parameters for the stimulation pulse signal are illustrated in Figure 2, and the adjustable ranges are listed in Table 1.

The electrical stimulation system comprises an implanted electrical stimulator and an external portable controller as shown in Figure 1. In addition to its basic function of generating stimulation pulses, the implanted electrical stimulator incorporated a pressure-detection module with a measuring capacity ranging from 0 to 250 kPa. The pressure on the GI tract was measured and transmitted outside in real time. When the device's power was lower than the minimum value, an alarm signal was transmitted to the external controller to inform the patient to switch on the external power transmission device. The external portable controller could set the stimulation parameters and display data from the internal device on the LCD. Meanwhile, pressure measurements would be recorded on the memory card for further study.

### 2.2. Transcutaneous Power Supply

In this study, the wireless power transmission system was based on the Ferrari electromagnetic induction laws. The transcutaneous power supply system comprised an in vitro power transmitter and an in vivo power receiver [15] as shown in Figure 1. The alternating magnetic field was the medium between the transmitter and receiver. The transmitting coil, driven by a square-wave current outside the body, generated varying magnetic fields. Based on inductive coupling, the receiving coil implanted under the skin induced AC voltage, which was then rectified and regulated into DC voltage to provide power to the in vivo electrical stimulator. The frequency of the alternating magnetic field was the same as that of the alternating current in the transmitting coil. In order to realize resonance, the inherent frequency of the receiving coil should also be the same as that of the alternating magnetic field.

#### 2.2.1. Transmission Efficiency

**Figure 3:**
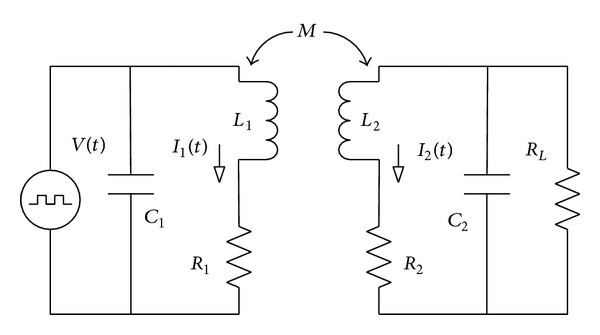
Power transmission link model.

To investigate the transmission efficiency of the wireless transcutaneous power supply system, a power transmission link model was developed and is shown in Figure 3.

In this figure, *L*_1_ and *L*_2_ are the self-inductances of the transmitting and receiving coils, respectively; *R*_1_ and *R*_2_ represent the winding resistances of the two coils; and *C*_1_ and *C*_2_ are the tuning capacitors. *R*_*L*_ is a resistor simulating the load, *M* is the mutual inductance between the two coils, and *V*(*t*) is a square-wave voltage source with frequency *f*.

The loop equation of the above-described circuit is given by

(1) $$\begin{array}{c} \left[\begin{array}{c} V\left(t\right)\\ 0 \end{array}\right]=\left[\begin{array}{cc} R_{1}+j\omega L_{1} & -j\omega M\\ -j\omega M & R_{2}+j\omega L_{2}+\frac{1}{1+j\omega C_{2}R_{L}} \end{array}\right]\left[\begin{array}{c} I_{1}\left(t\right)\\ I_{2}\left(t\right) \end{array}\right]. \end{array}$$

The determinant of the 2 × 2 matrix is

(2) $$\begin{array}{c} \Delta =\left|\begin{array}{cc} R_{1}+j\omega L_{1} & -j\omega M\\ -j\omega M & R_{2}+j\omega L_{2}+\frac{1}{1+j\omega C_{2}R_{L}} \end{array}\right|. \end{array}$$

The transmitting (3) and receiving (4) apparent power can be calculated as follows:

(3) $$\begin{array}{c} S_{t}=\left|\frac{V{\left(t\right)}^{2}\left(j\omega C_{1}\Delta +R_{2}+j\omega L_{2}+R_{L}/\left(1+j\omega C_{2}R_{L}\right)\right)}{\Delta }\right|, \end{array}$$

(4) $$\begin{array}{c} S_{r}=\frac{V{\left(t\right)}^{2}\omega^{2}M^{2}R_{L}}{\left|{\left(1+j\omega C_{2}R_{L}\right)}^{2}\right|\left|\Delta^{2}\right|}. \end{array}$$

Thus, the transmission efficiency can be written as

(5) η=(ω2M2RL)×((1+jωC2RL)2×|(jωC1Δ+R2+jωL2+RL/(1+jωC2RL))Δ|×|Δ2|)−1.

To maximize efficiency, the value of *C*_1_ and *C*_2_ must be carefully chosen to meet the criteria

(6) $$\begin{array}{c} \frac{\partial \eta }{\partial C_{1}}=0,\;\;\frac{\partial \eta }{\partial C_{2}}=0. \end{array}$$

The two coils should be designed to resonate with the same frequency *ω* to satisfy *ωL*_1_ = 1/*ωC*_1_ and *ωL*_2_ = 1/*ωC*_2_. Assume that the quality factors of the transmitting and receiving coils are *Q*_1_ = *ωL*_1_/*R*_1_ and *Q*_2_ = *ωL*_2_/*R*_2_, and the coupling coefficient is $k=M/\sqrt{L_{1}L_{2}}$.

When *Q*_2_^2^ = *Z*_*L*_/*R*_2_, the transmission efficiency can be calculated as

(7) $$\begin{array}{c} \eta =\frac{1}{4}k^{2}Q_{1}Q_{2}=\frac{M^{2}\omega^{2}}{R_{1}R_{2}}, \end{array}$$

which indicates that the efficiency can be optimized by promoting the resonant frequency, the mutual inductance, and the power factor.

Importantly, increasing the resonant frequency can promote transmission efficiency and reduce coil size. However, excessively high frequency can lead to intense skin and proximity effects. Additionally, the safety of the tissues around the receiving coil may be jeopardized. Thus, based on the experiments and considerations above, the resonant frequency of the transcutaneous power supply system was set at 230 kHz.

#### 2.2.2. Power Transmission Schematic

**Figure 4:**
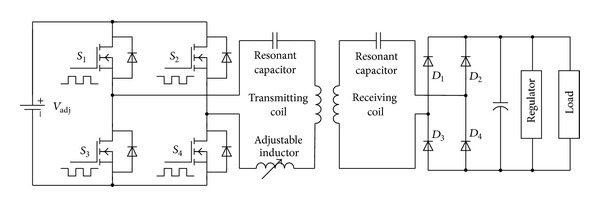
Schematic of the transcutaneous power supply system.

The power transmitter was composed of the wave generator and H-bridge energy emitter. The wave generator was designed to provide two opposite square-wave driven signals that would be fed to the H-bridge inverter to generate an alternating current in the LC tank, in which the wave frequency had been programmed to 230 kHz. The transmitting coil, a resonant capacitor, and an adjustable inductor were connected to form this LC tank. To maintain the transmitting circuit in a state of resonance with the driven signals' frequency, an adjustable inductor was added to eliminate the effect of environmental change on the LC tank. In the power receiver, the receiving coil had also been adjusted to resonate at 230 kHz. Four Schottky diodes were adopted as the rectifier and a capacitor was connected in parallel in order to smooth the rectified voltage. After being regulated, the power was input into the internal stimulator.  Figure 4 shows the schematic of the transcutaneous power supply system.

## 3. Experiments and Results

### 3.1. Test of the Electrical Stimulator

The internal stimulator was integrated into a box measuring 70 mm × 45 mm × 18 mm. Assuming five daily stimulation sessions (single-pulse stimulus mode, four-channel stimulation pattern with 16*V*_pp_ amplitude, 50% duty cycle, and 180 s duration per channel) and 500 Ω equivalent resistance of tissue, when using a Li-ion battery with voltage and capacity of 3.6 V and 1.3 Ah, the device life was calculated to exceed 4 months on a single charge. To protect the battery and extend service life, the charging voltage was set to 4 V, and the charging current was capped at 200 mA.

The performance of the GI electrical stimulation system was examined in a laboratory experiment. To simulate the variety of tissue during typical stimulation, resistive loads ranging from 200 Ω to 1 kΩ were connected to the output of each channel. Stimulation testing was performed using different combinations of parameters. In the air, the communication distance reached at least 3 meters.

### 3.2. Test of the Transcutaneous Power

A set of planar spiral coils were used in the transcutaneous power supply system. In order to maximize the receiving power, a disk-shaped magnetic core constructed from high-permeability MnZn ferrite was used to increase the magnetic flux, and AWG 40 enameled copper wire was wound around it to reduce the skin effect and proximity effect losses in the coils. An LCR tester (HIOKI 3532-50) was used to measure the electrical characteristics at a frequency of 230 kHz. The parameters of the transmitting and receiving coils are listed in Table 2.

**Figure 5:**
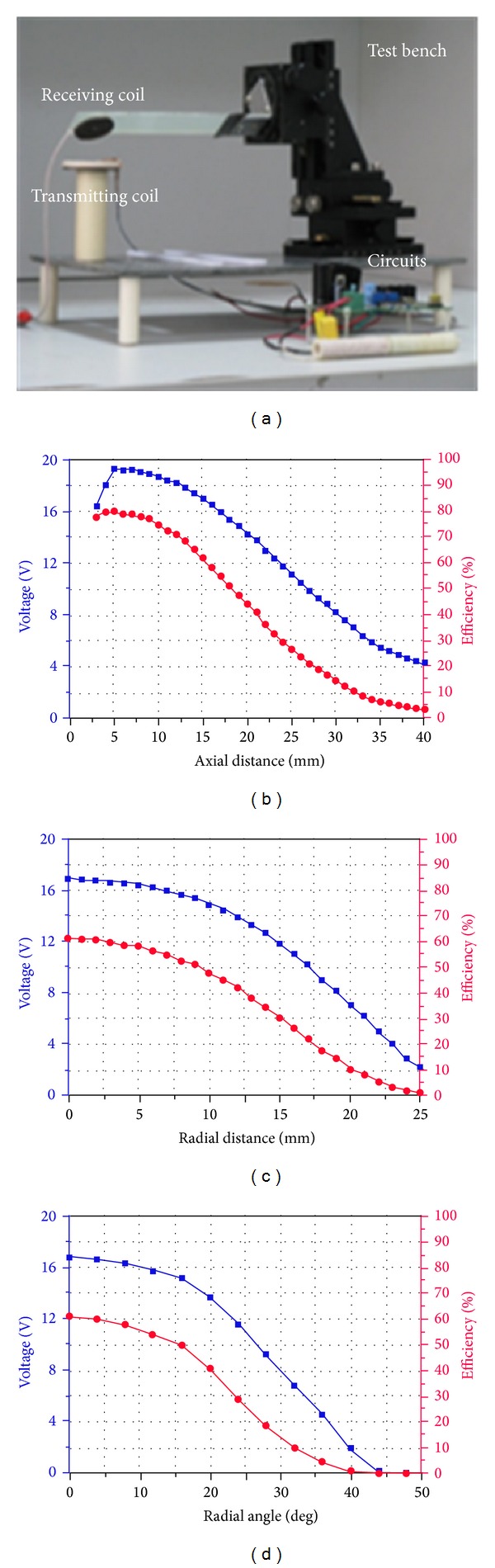
Test of transcutaneous power supply system. Test bench (a) and results in axial distance (b), radial distance (c), and radial angle (d).

The alignment of the transmitting and receiving coils and the distance between them would certainly vary during operation. Variable distance and misalignment of the coils can affect the coupling coefficient *k* and change the operating state of the inductive link. In order to investigate the performance of the transcutaneous power supply system, a test bench, as shown in Figure 5(a), was constructed.

1. The coils were set in the same axis, parallel to each other.  Figure 5(b) shows that the efficiency reached the maximum of 82% at a specific axial distance. When the axial distance was 15 mm, the efficiency and the voltage were 63% and 16.5 V, respectively.
2. The axial distance was fixed to 15 mm, and the radial distance changed from 0 to 25 mm. As shown in Figure 5(c), when the radial distance increased from 0 to 10 mm, the transmission efficiency and the output voltage decreased slightly. The efficiency was still about 50%, and the output power was more than 780 mW when the radial distance was 10 mm.
3. In Figure 5(d), the results were measured by pivoting the receiving coil 4 degrees each time. When the radial angel was less than 15 degrees, the transmitting efficiency was up to 50%, and the output power was more than 1 W.

### 3.3. Animal Experiment

**Figure 6:**
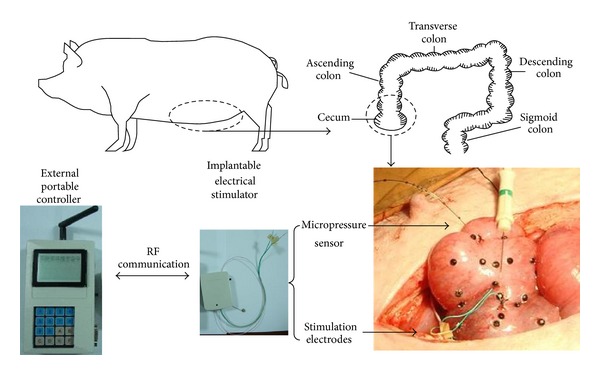
Animal experiment.

Two healthy pigs weighing 42 ± 3.8 kg and two STC pigs weighing 40 ± 2.9 kg were selected in the animal experiment. The pigs were fasted for 24 hours before surgery, but water was given to promote emptying of the colon. Each pig was premedicated with intramuscular ketamine (20 mg/kg) and diazepam (2 mg/kg). General anaesthesia was maintained with pentobarbital (5%) by intravenous injection as appropriate. Saline and glucose were continuously transfused at a 1 : 1 ratio with 10 mL/kg/h and an air-oxygen mixture was supplied by orotracheal intubation. Vital signs were monitored by medical devices. In the experiment, each pig's abdomen was opened with a median incision of 20 cm, and the cecum was fully exposed. From the blind end of the cecum, 4 pairs of 3 mm long and 0.2 mm diameter stainless steel stimulation electrodes were sutured into the seromuscular layer of the cecum at a distance of 3 cm. Peristalsis mark points were placed on the surface of the cecum in equal intervals of 1 cm. At a distance of 10 cm from the blind end of the cecum to the ileocecal valve, a 0.4 cm incision was done, and the pressure sensor was inserted into the cavity of the cecum. The internal devices were sealed with medical silicone. The animal experiment operation is shown in Figure 6.

After the electrical stimulator was implanted in the pigs, the communication was unobstructed in the range of 1.5 m. A myoelectricity tester (Medtronic Keypoint 4) was attached to the smooth muscle of the cecum to monitor the myoelectricity variation that was brought about by the stimulation. Different trains of rectangular impulses were produced to stimulate the seromuscular layer for 20 seconds. Shrinkage of about 30% was adopted as standard to evaluate the stimulation parameters [8]. To reduce the fatigue of the cecum, each group of impulses was performed in triplicate and there was a 3-4 minute interval between stimulations.  Table 3 lists the cecal reactions to different stimulation parameters; the incubation was counted after the stimulation.

When testing contractions produced in the STC pigs, low-intensity stimuli (*V*_pp_ = 7.5 V, *T*_*w*_ = 0.3 ms, *f* = 10 Hz) failed to generate visible contractions regardless of the duration of the applied stimuli. With increasing stimulation intensity, it was obvious that the ceca of the healthy pigs were more sensitive than those of the STC pigs. The results proved that electrical stimulation can promote cecal contraction and that different stimulation parameters had varied effects on the contraction incubation and intensity. By comparison, the frequency *f* mainly affected the stimulation incubation, the pulse width *T*_*w*_ primarily influenced the stimulation intensity, and *V*_pp_ had the greatest effect on both the intensity and incubation.

After pacing the pulse width in the range of 3–5 ms and setting the voltage and frequency at 10 V and 40 Hz, respectively, the response of the smooth muscle was found to be always visible between and slightly beyond the stimulating electrodes in both the healthy and STC pigs. Contractions that were induced by stimulation involved shortening in both longitudinal and radial directions. As we observed in the experiment, in Table 3, the parameters of 50 Hz, 20 V amplitude, and 3 ms pulse width evoked a corresponding longitudinal shortening of about 30%. But higher intensity tended to generate significantly stronger contractions until the tetanic contraction occurred in the healthy pigs when *V*_pp_ = 12 V, *T*_*w*_ = 5 ms, and *f* = 120 Hz.

**Figure 7:**
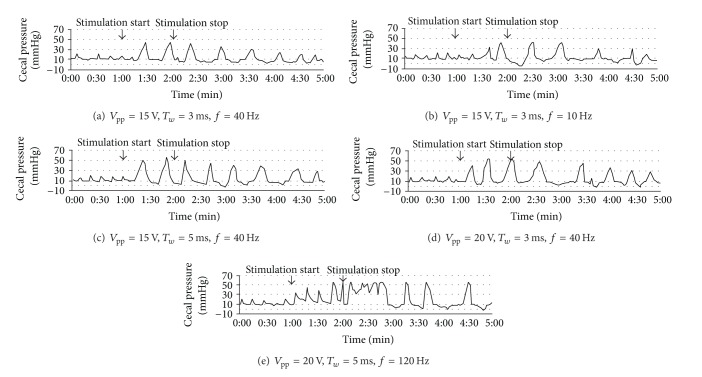
Cecal pressure of an STC pig under different stimulation parameters.

To further investigate the stimulation reactions of the STC pigs, additional intensity stimuli were performed and more detailed pressure data were recorded. The STC pig experiment was conducted as follows: (1) cecal pressure data were collected for 1 min before stimulation; (2) the cecum was stimulated for 1 min and the pressure signal was monitored; (3) cecal pressure data were collected for 3 min after stimulation. The stimulator parameters were selected to be a peak-to-peak voltage of 15 V and 20 V, with frequencies of 10 Hz, 40 Hz, and 120 Hz and pulse widths of 3 ms and 5 ms.  Figure 7 illustrates the detailed pressure variation of an STC pig's cecum under different stimulation parameters. When comparing (a) with (b), (c), and (d), the waveform generated by varied pressure verified the impact of the parameters on the electrical stimulation. During operation, the stimulation pulses did not visibly damage the tissue. However, when stimulation parameters reached a 5 ms pulse width, 120 Hz frequency, and 20 V peak-to-peak voltage when the power was at a high level, the cecum remained in a tetanic contraction as shown in Figure 7(e) and injury would occur.

**Figure 8:**
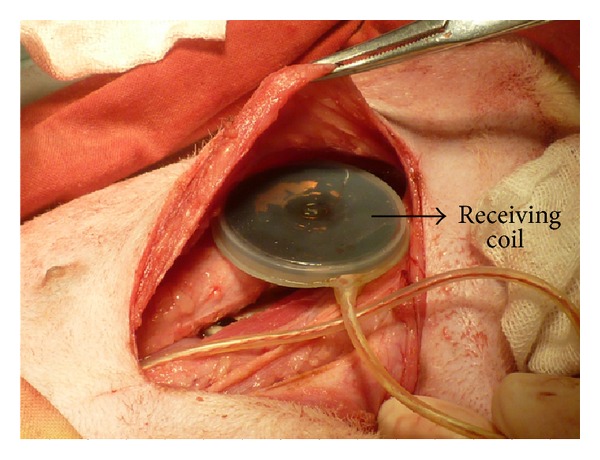
Implantation of the receiving coil.

After considering the transmission range of the transcutaneous power supply system, the receiving coil was connected to the internal stimulator and implanted under the skin as shown in Figure 8. An electrical stimulator with an operational charging alarm was used to test the performance of the transcutaneous power supply system. Given a maximum charging power of 800 mW, the transmitting current was set to 200 mA, which meant that the transmitting power was 2 W and the transmitting coil was fixed optimally to the receiving coil. In the end, the charging operation lasted about 6 hours until the alarm signal indicated that the device had been fully charged.

## 4. Discussion and Conclusion

The aim of this study was to assess the feasibility of and methodology behind inducing reproducible contractions of the cecum with the proposed GI electrical stimulation system and wireless transcutaneous power transmission technology to resolve the bottleneck of limited battery power and realize permanent implantation.

The electrical stimulator was capable of producing ideal stimulation pulses, and the parameters could be programmed by the external portable controller in wide ranges via wireless communication. Amaris et al. published the emptying measurements on anaesthetized dogs by using rectangular trains of 50 Hz, 20 V amplitude, 10 ms pulse width, and 18 s duration. After the stimulation, 78% of the pellets emptied [7]. Bertschi et al. proposed the stimulation parameters of 10 V amplitude over 30 s, with a pulse width of 1 ms and a frequency of 120 Hz with a similar experimental setup on anaesthetized pigs, which induced a corresponding longitudinal shortening of about 30%. However, the battery-operated stimulator was limited to a maximum pulse width of 1 ms and maximum amplitude of 10 V; thus, exploring various parameters' effects on inducing contractions was limited [8]. In this in vivo experiment, we used different trains of bipolar, rectangular voltage that effectively induced cecal contractions and measured variations among different parameters (incubation, pressure, etc.) to demonstrate the responses in healthy and STC pigs. Powerful, ring-like contractions that propagated in peristaltic fashion with each instance of stimulation were achieved without signs of muscular fatigue or tissue damage. An optimal stimulation protocol is proposed: stimulation bursts of 3 ms pulse width, 10 V amplitude, 40 Hz frequency, and 20 s duration which induced cecal shrinkage of about 30% in both healthy and STC pigs. When stimulation energy reached a specific threshold (whether voltage, frequency, or pulse width increases), GI motility ceased to improve; rather, these excessive energy expenditures caused a tonic spasm and damage to the GI tissue. For this reason, the stimulation parameters for an FGID patient should not exceed 5 ms pulse width, 20 V amplitude, and 120 Hz frequency. Although, in theory, contractions of the GI tract are modulated by the nervous system and the anesthesia has limited influence on the stimulation effect, the experiment should be replicated with conscious pigs to clarify this issue.

Pressure detection provided feedback for the stimulation system. In clinical practice, pressure data could be used to test whether a particular patient might benefit from stimulation. When considered with clinical symptoms, the appropriate stimulation parameters could be obtained for the patient.

Adopting transcutaneous power paved the way for permanent implantation of gastric electrical stimulation devices. This work investigated different coil alignments to optimize the device's power supply. In the animal experiment, 6 hours were needed to fully charge the battery wirelessly (through the biological tissue). The distance between the transformer windings would be approximately equal to the thickness of the patient's skin, fatty tissue, and muscle: usually between 1 and 2 cm [16]. The receiving power could be guaranteed by increasing the transmitting current. However, the effect that prolonged exposure to an intense magnetic field would have on human tissue should be studied more thoroughly to ensure that doing so would not jeopardize patient safety.

Evaluating the long-term effects of this technique on the tissue surrounding the stimulating electrodes, accommodation, and absorption is necessary before these technologies can be fully integrated into clinical practice. Possible secondary effects (e.g., pain) must also be studied before applying this method to humans.

In conclusion, a GI electrical stimulation system based on transcutaneous power transmission technology has been proposed; this system employed an external portable controller (to program sequential stimulation with adjustable parameters) to artificially produce and propagate contractions in peristaltic fashion. Real-time pressure detection monitored the reaction of the GI tract. Transcutaneous energy transmission technology ensured that the power supply would be adequate for long-term implantation, which, in turn, will improve patient quality of life and reduce the risks associated with additional procedures to refresh the power supply.

## Figures and Tables

**Table 1:** Stimulation parameters.

Parameters	Parameters' range	
	Unit	Range
Amplitude (peak-peak)	V	8–25
Pulse cycle	ms	0.1–999
Pulse width	ms	0.1–999
Stimulation duration	s	1–999

**Table 2:** Parameters of transmitting coil and receiving coil.

Parameters	Radius (mm)	Height (mm)	Inductance (*μ*H)	Capacitance (nF)	Impedance (Ω)	*Q*
Transmitting coil	60	5.6	251.0	1.9	2.0	235.0
Receiving coil	35	3	140.0	3.4	4.6	44.0

**Table 3:** Reactions of the cecum to different stimulation parameters.

Stimulation parameters			Healthy pig				STC pig			
*V*_pp_ (V)	*T*_*w*_ (ms)	*f* (Hz)	Incubation (ms)	Myoelectrical activity	Pressure rise (mmHg)	Shrinkage (%)	Incubation (ms)	Myoelectrical activity	Pressure rise (mmHg)	Shrinkage (%)
7.5	1	10	16.1 ± 2.1	6.2 ± 1.8	5.2 ± 1.1	8.2 ± 2.26	21.1 ± 2.3	2.1 ± 0.4	3.2 ± 0.5	7.3 ± 1.19
7.5	3	10	15.2 ± 3.5	14.1 ± 3.2	13.6 ± 2.7	14.5 ± 3.15	19.8 ± 3.7	5.4 ± 1.6	6.6 ± 2.1	6.4 ± 1.41
7.5	5	10	14.1 ± 2.4	22.9 ± 4.1	24.1 ± 2.3	18.7 ± 3.71	18.8 ± 3.5	8.3 ± 1.2	13.9 ± 0.8	12.6 ± 2.48
10	0.1	40	9.5 ± 1.2	8.3 ± 1.5	9.8 ± 1.1	11.4 ± 2.49	15.1 ± 2.5	4.8 ± 1.2	5.2 ± 1.0	4.4 ± 1.52
10	0.3	40	9.3 ± 2.1	12.1 ± 2.8	9.4 ± 1.4	15.7 ± 2.41	14.6 ± 1.9	7.5 ± 2.3	3.7 ± 0.6	8.3 ± 2.09
10	1	40	9.0 ± 1.6	27.8 ± 4.3	12.6 ± 2.4	15.3 ± 4.29	12.1 ± 1.1	13.4 ± 1.7	5.5 ± 0.7	11.6 ± 2.84
10	3	40	8.8 ± 0.9	29.2 ± 7.8	27.5 ± 3.4	31.4 ± 4.44	13.4 ± 0.9	15.3 ± 1.7	22.8 ± 5.0	29.4 ± 3.82
10	5	40	8.3 ± 1.1	36.5 ± 11.2	37.4 ± 4.2	38.4 ± 6.56	12.5 ± 1.1	18.5 ± 3.1	36.0 ± 5.4	32.4 ± 4.72
10	10	40	7.9 ± 0.8	47.6 ± 7.5	59.5 ± 4.7	40.7 ± 7.01	11.0 ± 1.4	37.5 ± 8.1	44.1 ± 2.9	39.4 ± 8.82
12	0.1	120	5.1 ± 0.8	11.7 ± 1.8	20.0 ± 1.7	14.6 ± 4.32	12.5 ± 0.8	12 ± 0.5	12.6 ± 1.9	9.3 ± 2.19
12	0.3	120	4.2 ± 0.6	17.3 ± 2.1	34.2 ± 2.3	21.9 ± 6.17	9.3 ± 0.8	11.4 ± 1.3	12.8 ± 1.1	11.4 ± 2.42
12	1	120	4.1 ± 0.4	38.3 ± 4.2	51.9 ± 4.7	42.7 ± 8.81	8.8 ± 1.3	18.5 ± 2.1	36.1 ± 2.1	36.7 ± 7.01
12	3	120	3.8 ± 0.3	41.7 ± 5.1	60.1 ± 5.5	44.4 ± 9.32	8.9 ± 2.1	31.3 ± 7.9	43.2 ± 3.0	38.5 ± 11.75
12	5	120	3.6 ± 0.2	Tetanic contraction	71.4 ± 1.3	46.8 ± 11.04	5.6 ± 0.8	43.7 ± 11.2	43.1 ± 5.3	39.6 ± 12.02

*The unit of myoelectrical activity is times per 5 min which is counted after the stimulation.
